# Critical Role of E1623 Residue in S3-S4 Loop of Nav1.1 Channel and Correlation Between Nature of Substitution and Functional Alteration

**DOI:** 10.3389/fnmol.2021.797628

**Published:** 2022-01-10

**Authors:** Tao Su, Meng-Long Chen, Li-Hong Liu, Hen Meng, Bin Tang, Xiao-Rong Liu, Wei-Ping Liao

**Affiliations:** ^1^Department of Neurology, Institute of Neuroscience, Second Affiliated Hospital of Guangzhou Medical University, Guangzhou, China; ^2^Key Laboratory of Neurogenetics and Channelopathies of the Ministry of Education of China, Guangzhou, China

**Keywords:** missense, sodium channel, SCN1A, epilepsy, prediction

## Abstract

**Objective**: An overwhelming majority of the genetic variants associated with genetic disorders are missense. The association between the nature of substitution and the functional alteration, which is critical in determining the pathogenicity of variants, remains largely unknown. With a novel missense variant (E1623A) identified from two epileptic cases, which occurs in the extracellular S3-S4 loop of Na_v_1.1, we studied functional changes of all latent mutations at residue E1623, aiming to understand the relationship between substitution nature and functional alteration.

**Methods**: Six latent mutants with amino acid substitutions at E1623 were generated, followed by measurements of their electrophysiological alterations. Different computational analyses were used to parameterize the residue alterations.

**Results**: Structural modeling indicated that the E1623 was located in the peripheral region far from the central pore, and contributed to the tight turn of the S3-S4 loop. The E1623 residue exhibited low functional tolerance to the substitutions with the most remarkable loss-of-function found in E1623A, including reduced current density, less steady-state availability of activation and inactivation, and slower recovery from fast inactivation. Correlation analysis between electrophysiological parameters and the parameterized physicochemical properties of different residues suggested that hydrophilicity of side-chain at E1623 might be a crucial contributor for voltage-dependent kinetics. However, none of the established algorithms on the physicochemical variations of residues could well predict changes in the channel conductance property indicated by peak current density.

**Significance**: The results established the important role of the extracellular S3-S4 loop in Na_v_1.1 channel gating and proposed a possible effect of local conformational loop flexibility on channel conductance and kinetics. Site-specific knowledge of protein will be a fundamental task for future bioinformatics.

## Introduction

The distinct function of a protein depends on the well-organized composition of the amino acids and intricate folding of the molecule. Gene mutations result in either gross protein malformation (referred to destructive variants), that mostly led to haploinsufficiency, such as truncation and splice-site (He et al., 2019), or residue substitution (missense). A part of genetic defects may translate into functional alterations and subsequently lead to human diseases. Advances in genetic sequencing technology have enabled the detection of numerous sequence variants in human beings. An overwhelming majority of the variants are missense (gnomAD, HGMD), which result in the substitution of the amino acid (AA) at residues. In contrast to destructive mutations that result in remarkably damaging effects on protein composition, the functional consequence of missense mutations is far more unpredictable. Our previous study demonstrated that the molecular sub-regional location of the variant plays a critical in determining the damaging effect of the mutations (Tang et al., 2020). However, the pathogenicity or functional impact of a missense mutation depends not only on its location but also potentially on the nature of amino acid substituted, such as molecular mass, polarity, and acidity. The association between the nature of substitution and the functional alteration, which remains largely unknown, is a critical aspect in exploring the molecular mechanism underlying the pathogenicity of variants.

Genetic defects in voltage-gated sodium channel (Na_v_1.1) α subunit encoded by *SCN1A* are a major cause of epilepsy. To date, more than 1,700 *SCN1A* variants have been reported to be associated with epilepsy and other episodic disorders^1^ (Meng et al., 2015). The *SCN1A* associated epilepsies compose a wide spectrum of phenotype ranging from milder febrile seizures to severe epileptic conditions, typically Dravet syndrome (DS; Claes et al., 2003; Nabbout et al., 2003; Ceulemans et al., 2004; Mulley et al., 2005; Harkin et al., 2007; Gambardella and Marini, 2009; Dravet and Oguni, 2013; Kasperaviciute et al., 2013; Gataullina and Dulac, 2017). Previous studies have demonstrated that DS or severe epilepsies are associated with destructive variants such as nonsense and frameshift variants, or missense in the pore-region that caused loss-of-function of Na_v_1.1. Although missense variants in other regions of Na_v_1.1 have also been reported (Meng et al., 2015), their functional relevance has not been well studied. Factors that determine the pathogenicity of *SCN1A* missense variants remain unclear.

In this study, we identified a novel distinct missense *SCN1A* variant (c.4868A>C/E1613A) in two cases with epilepsy and febrile seizures. The substitution occurs at the putative extracellular loop linking S3 and S4 in DIV. The functional role of the extracellular S3-S4 loop remains largely unknown, except that a few studies identified toxin binding sites within the loops (Rogers et al., 1996; Bosmans et al., 2008; Wang et al., 2011). To dig deep into the functional role of E1623 and the factors that influence the pathogenicity of missense variants, six E1623 mutants with all possible substitutions caused by single nucleotide variations (SNV) were artificially created using site-directed mutagenesis. We determined the electrophysiological properties of these mutants and analyzed the correlations between the residue properties and electrophysiological alterations.

## Materials and Methods

### Patient and Genetic Analysis

Diagnoses and treatments of the patients were conducted in the epilepsy center of the second affiliated hospital of Guangzhou Medical University. Clinical data including medical records, standardized questionnaires, and EEG recordings were collected. The probands and relevant familial members were assessed for genetic variations using a standardized protocol after providing written informed consent. This study was approved by the Research Ethics Board of the Hospital. Genomic DNAs were prepared from ethylene diaminetetraacetic acid-treated whole blood samples. The *SCN1A* mutation was identified by direct *SCN1A* screening as in our previous report (Liao et al., 2010). The *SCN1A* mutations were described according to the nomenclature established (den Dunnen and Antonarakis, 2000), and numbering was started from the initiating ATG codon.

### Bioinformatics

Na_v_ channel nucleotide sequences were derived from the NCBI database^2^. The NCBI database was queried with amino acid sequences of human Na_v_1.1 to obtain the corresponding information of DNA locus and related functional regions. To determine whether other amino acid substitutions occur in the vicinity of the mutation site, information of SNPs and mutations were queried from the SNP database of the NCBI and our *SCN1A* mutation database^1^. All amino acid sequences of the Na_v_ families were retrieved from GenBank and saved as individual FASTA formatted files. The sequences including the S3-S4 region of DIV were subjected to multiple sequence alignment analysis by Clustal Omega with a few modifications in the color grouping. The sequence ranges of specific S3-S4 regions are in accord with that is annotated for human Na_v_1.1 isoform1 in the NCBI database.

The high resolution cryogenic electron microscopy structure of human Na_v_1.1 (PDB ID: 7DTD, 3.3 Å taken from the protein data bank^3^) was used as a template for subsequent modeling (Pan et al., 2021). Three-dimensional (3D) modeling of the human wild-type (WT) and mutant Na_v_1.1 was performed using SWISS-MODEL, an automated homology modeling program^4^.

The predicted effects of all latent amino acid substitutions at E1623 were scored by different established predictive tools based on different substitution score systems, including SIFT, Mutationassessor, PolyPhen, PROVEAN, I-MUTANT suite, SNAP2, and STRUM (Tang et al., 2020).

### Mutagenesis and Heterologous Expression

Plasmids containing WT full-length sequence of human Na_v_1.1 alpha subunit (pCMV-*SCN1A*) and beta subunit 2 (pGFP-IRES-*SCN2B*) was kindly provided by professor Alfred L Jr George (Lossin et al., 2003). A full-length sequence of beta subunit 1 was constructed into a vector driving the bicistronic expression of red fluorescent protein (pDsred-IRES-*SCN1B*) in our lab. Site-directed mutagenesis at E1623 was performed using the Quick Change XL site-directed mutagenesis kit (Stratagene, La Jolla, CA) according to the manufacturer’s protocols. All mutations were confirmed by DNA sequencing of the region surrounding the mutation. HEK 293T cells were grown in 1:1 Ham’s F-12 and Dulbecco’s modified eagle’s medium (DMEM) supplemented with 10% fetal bovine serum, 100 U/ml of penicillin, and 100 μg/ml streptomycin. The cells were maintained in a humidified incubator at 37°C with 5% CO_2_. Cells were then co-transfected with pCMV-*SCN1A*, and pDsred-IRES-*SCN1B* and pGFP-IRES-*SCN2B* plasmids (5 μg each) using Lipofectamine 3000 reagent Kit from Thermo Fisher Scientific. After incubation for 12–15 h, cells were replated in 35-mm culture dishes.

### Electrophysiological Recordings and Analysis

Electrophysiological studies were performed 20–48 h after transfection, according to our previous report (Chen et al., 2015). Channel activity was recorded by using the conventional patch clamp technique in the whole-cell configuration with an Axopatch 200B patch-clamp amplifier (Axon Instruments). The extracellular control solution contained: 140 mM NaCl, 3 mM KCl, 1 mM CaCl_2_, 1 mM MgCl_2_, 10 mM HEPES, and 5 mM glucose, adjusted to pH 7.4, 310 ± 6 mOsm. The whole-cell pipette solution contained: 140 mM CsF, 1 mM EGTA, 10 mM HEPES, and 10 mM NaCl, pH 7.4, adjusted with CsOH, 310 ± 6 mOsm. Cell capacitance was calculated by integrating the area under an uncompensated capacity transient elicited by a 10-mV hyperpolarizing test pulse from a holding potential of −80 mV. Series resistance was compensated by at least 70% in all recordings. Leakage currents were subtracted by the P/N method. The pCLAMP 10.2 software (Axon Instruments) was used for the acquisition and analysis of currents. Current amplitude in response to each test pulse was normalized to the maximum. The voltage dependence of activation and inactivation was determined using standard protocols. The conductance (G) was calculated according to: G = I_peak_/(V_test_ − V_rev_), where V_rev_ is the Na^+^ reversal potential, V_test_ is the command potential, and I_peak_ is the peak current amplitude. G/G_max_ was then fitted with the following Boltzmann equation: G/G_max_ = (1 + exp((V_m_ − V_1/2_)/k))^−1^, where G_max_ is the maximal conductance, V_1/2_ is the half-activation potential, V_m_ is the test voltage, and k is the slope factor. From this equation, we derived parameters of the midpoint (V_1/2_) and slope factor of steady-state activation and inactivation curves. Parameters for recovery of channels from fast inactivation were determined using a double pulse protocol. Channels were inactivated with −10 mV conditioning pulse for 100 ms, followed by command hyperpolarization for varying durations as a recovery period (1 ms to 1 s), and subsequent test pulse to −10 mV (for 10 ms). The normalized recovery curve was fit with a double-exponential function to obtain tau for recovery: I/I_max_ = A1×(1 − exp(−t/τ-fast)) + A2×(1 − exp(−t/τ-slow)), where I is the current amplitude at the time point t after the onset of the voltage command, I_max_ is the maximal recovery current amplitude, and A is the amplitude contribution of the different recovery time constants. Time constants of recovery τ-fast and τ-slow were extracted from the equation as recovery parameters.

### Statistical Analysis

Data reported throughout the text and figures are presented as means ± SEM. Statistical analyses were conducted in SPSS 19.0, using Student’s *t*-test when comparisons were made between two groups or by one-way ANOVA with a *post hoc* Tukey HSD test for comparing data from multiple groups. Significance was assigned at *P* < 0.05.

## Results

### Epilepsy-Associated Variant E1623A and the Extracellular Loop

**Figure 1:**
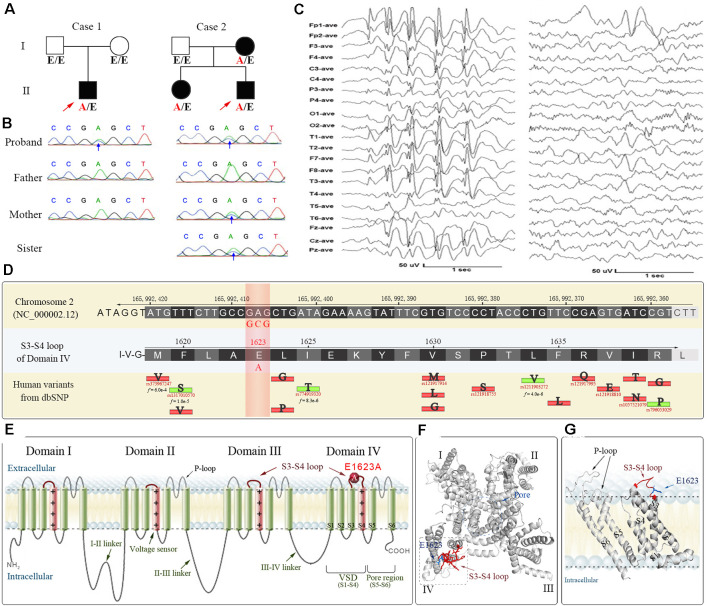
The pedigrees, DNA chromatograms, EEG, and bioinformatical annotations of the E1623A variant of *SCN1A* gene identified from two cases of epilepsy. **(A)** Pedigrees of two cases. Case 1 was a 17.5-year-old boy with Dravet syndrome. The proband of case 2 was diagnosed as epilepsy with febrile seizure plus. His mother and elder sister had several febrile seizures before 6 years old. A/E indicates heterozygous E1623A carrier, while E/E indicates wildtype. **(B)** Sanger sequencing verification. **(C)** Representative EEG of the case 1 proband showing the generalized high amplitude sharp and slow waves (left), along with focal sharp and slow waves in the left frontal and temporal regions (right). **(D)** Collection of reference sequences and human variants within the S3-S4 extracellular loop of DIV where the E1623A occurred. The position at E1623 has not been reported with any variant according to human databases. The collected missense variants with epilepsy or non-clinical implications are indicated by red or green bars with their substituted AA abbreviations. Available minor allele frequency (*f* value) is under the reference SNP IDs. **(E)** Structure-function map of Na_v_1.1 indicating the location of E1623A. **(F)** A top view of 3D structural modeling of the Na_v_1.1 alpha unit shows that the S3-S4 loop and E1623 were at a distance from the central pore region. **(G)** A side view of the arrangement of the VSD highlights the extracellular S3-S4 loop and the location of E1623. The S4 helices are depicted in pink, while other helices are in green. The S3-S4 loop is highlighted in red.

An *SCN1A* variant c.4868A>C/p.E1623A was identified from two cases of epilepsy, including a case with de novo variant and a familial case with three individuals affected (Figures 1A–C). Case 1 was a 17.5-year-old boy with Dravet syndrome. The proband of case 2 was diagnosed as epilepsy with febrile seizure plus. The proband’s mother and elder sister had several febrile seizures before 6 years old (clinical information refers to Supplementary Data). The variant occurs in exon 26 of *SCN1A* and leads to the substitution of glutamic acid by alanine at E1623 that locates in the extracellular loop linking S3-S4 in DIV of Na_v_1.1 (Figures 1D,E). The E1623A is a novel missense variant despite several disease-associated or low-frequency variants flanking the site of E1623. The 3D structural modeling showed that E1623 residue and its correlated loop were located on the top of the lateral voltage-sensor domain (VSD, S4; Figures 1F,G).

**Figure 2:**
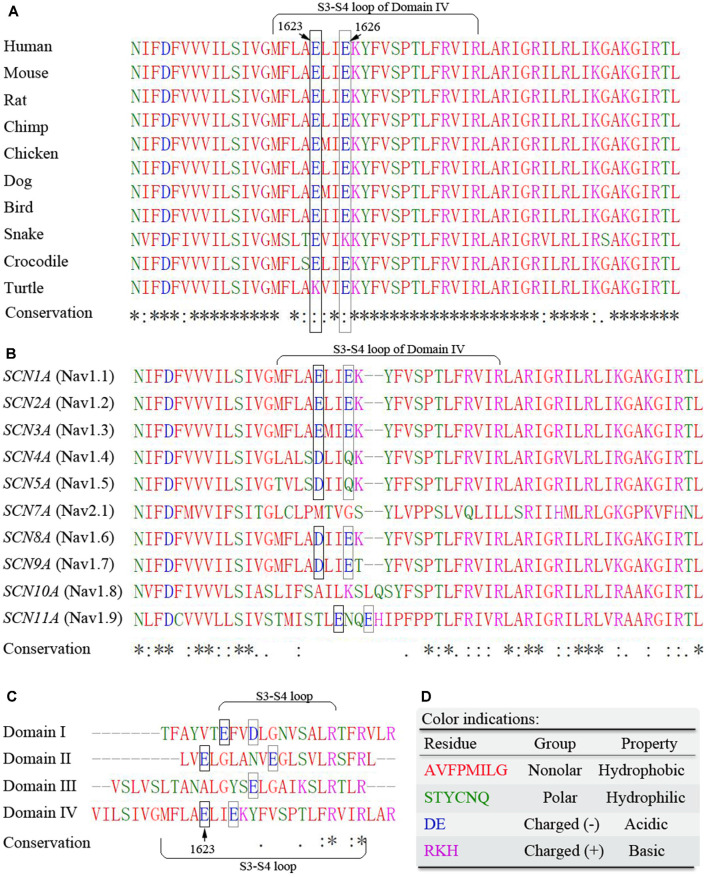
Multiple sequence alignments of the S3-S4 extracellular loop of DIV. **(A)** Cross-species alignments show high conservation of amino acid sequence within the region. **(B)** Sequence alignments among Na_v_ family members indicate high conservation of two acidic residues E1623 and E1626. **(C)** Differences in sequence length and residues of the S3-S4 loop among different domains of human Na_v_1.1 alpha unit. **(D)** Color indications. Residues are grouped by their physicochemical properties and indicated by different colors. The symbols of conservation significance are in keeping with the Clustal Omega from the NCBI website. * fully conserved; : conservation with strongly similar properties; . conservation with weakly similar properties; blank space indicates not conserved. The potentially important acidic residues are highlighted with frames.

Sequence alignments for conservation showed that the amino acid sequence of the S3–S4 loop in DIV of Na_v_1.1 is highly conserved across species. It is noteworthy that a potential motif constituted by two residues at positions E1623 and E1626 remains evolutionarily conserved in their negative charged property (Figure 2A). Residues from E1623 to E1626 form a tight turn of the loop on the top of the lateral VSD (Figure 1G). Among different alpha subunits of Na_v_ family that contribute to different Na_v_ properties, the sequence is slightly variable, but conserved with two alternative acidic residues, glutamate or aspartate, at the equivalent position as E1623 in Na_v_1.1 (Figure 2B). The other three asymmetric domains (DI-III) of human Na_v_1.1 also showed conservation of the glutamate residue, even though they have the much shorter S3-S4 loop (Figures 2C,D).

### Consequences of Substitutions at E1623

**Figure 3:**
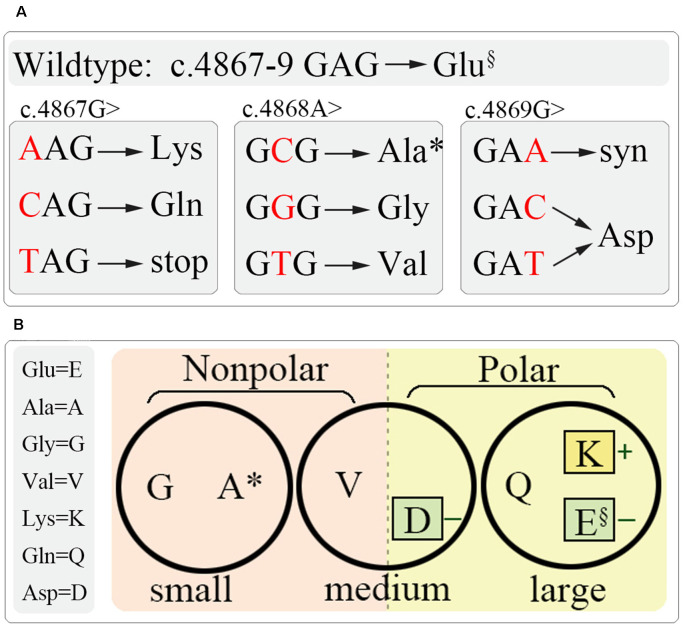
Schematic diagram of site-directed mutagenesis at E1623. **(A)** All latent variants by single nucleotide variation at c.4867–4869. **(B)** Residue grouping according to their basic physiochemical properties, including molecular weight (circles), polar (colored squares), and charge (little colored squares). §, wild-type; *, the identified mutation.

To understand the role of this potentially important glutamate residue on channel function and the link between residue property and functional alteration, we generated all the latent substitutions at residue E1623 caused by SNVs. The substitutions included alanine (A), aspartic acid (D), glycine (G), lysine (K), glutamine (Q), and valine (V). These residues together with glutamate happen to show a spectrum of residues with different properties, such as molecular mass, polarity, and charge properties (Figure 3).

**Figure 4:**
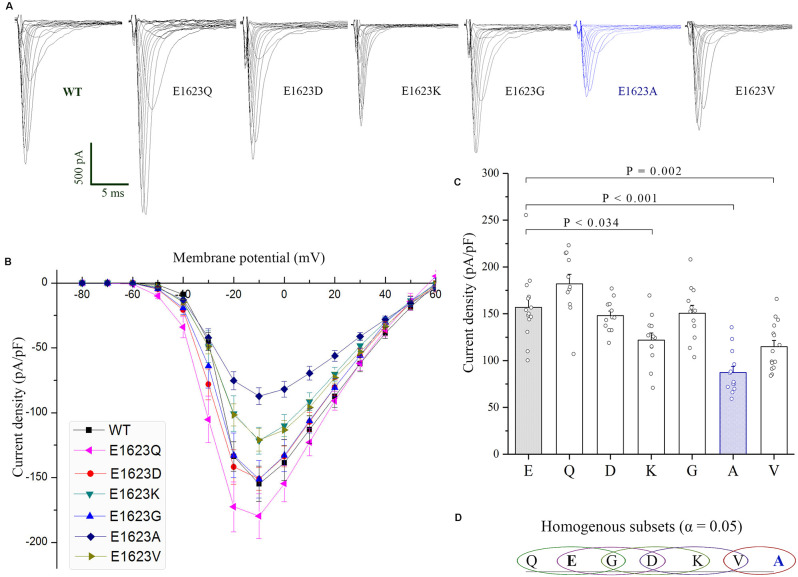
Current densities of E1623 mutants. All variants were expressed in HEK293T cells *via* transfection. **(A)** Representative current traces elicited with 20-ms depolarizations ranging from −80 mV to +60 mV in +10 mV steps. Intervals between pulses were 10 s. The mutants were arrayed by their residue similarity to WT according to Grantham’s distance. Currents are noticeably reduced in the E1623A variant. **(B)** Plots of current densities and membrane potentials. **(C)** Group data of peak current densities elicited at −10 mV. **(D)** Statistical output of homogenous subsets shows significant differences at the 0.05 level among the variants, *via* one-way ANOVA and *post hoc* Tukey HSD tests.

The WT and diverse mutant alpha-subunits, which were produced by site-directed mutagenesis, were expressed transiently in HEK293T cells. We first examined the plasma membrane expression of these mutants by Western blot and did not find any significant difference among the mutants (Supplementary Data). Subsequently, the mutants were subjected to examine their functional effects by using whole-cell voltage clamp recording. These mutants manifested many different amplitudes of Na^+^ currents (Figure 4A). The peak current density of the epilepsy-associated mutant E1623A was significantly decreased (*P* < 0.05; Figures 4B–D), while the mutant E1623Q was slightly increased when compared with that of WT, but did not reach statistical significance (*P* > 0.05). It was noted that E1623K and E1623V also resulted in a significant decrease in current density (*P* < 0.05).

**Figure 5:**
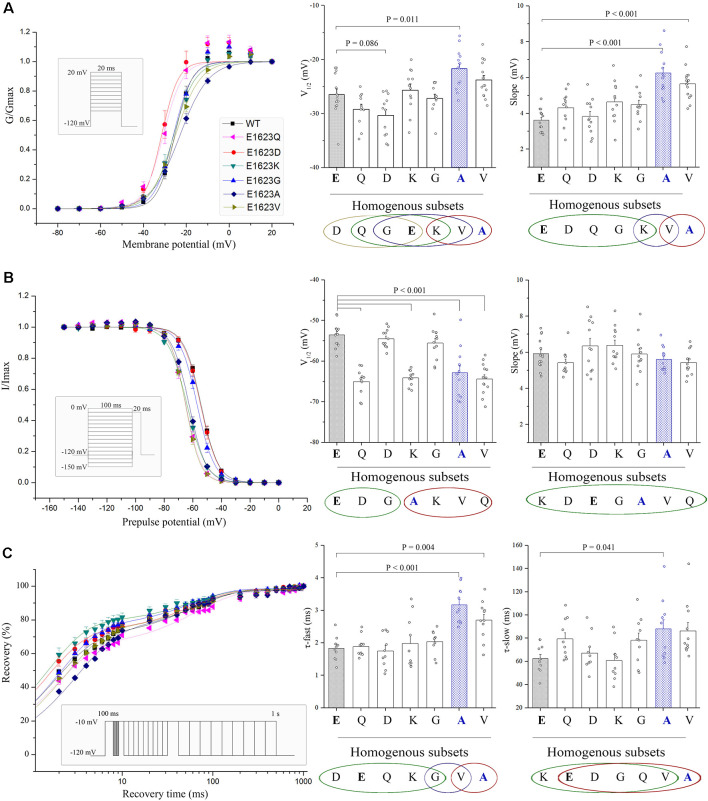
Voltage dependence and recovery kinetics of the E1623 mutants. **(A)** Activation curves of all E1623 mutants. **(B)** Steady-state inactivation curves. For testing voltage dependent channel availability in fast inactivation, a two-pulse protocol was used. Na^+^ currents were evoked by a 20-ms test pulse to −10 mV after 100-ms conditioning prepulses between −150 and 0 mV in 10-mV increments. Peak currents evoked by test pulses were measured, normalized, and plotted against the conditioning prepulse potentials. Parameters of activation and fast inactivation were estimated by fitting current-voltage relationships with the Boltzmann function. Estimated parameters including half-activation potential (V_1/2_) and slope factor, were derived from the Boltzmann fits. Group data of the V_1/2_ and slope are shown at the right panels. **(C)** Recovery from fast inactivation in paired stimulus experiments. Paired stimuli were applied at various inter-stimulation intervals. The recovery fractions were plotted to the intervals. The normalized recovery plots were fit with a double-exponential function to obtain parameters of τ-fast and τ-slow (shown in right panels). Statistical outputs of homogenous subsets (shown below the plots; those within the same circle are *α* > 0.05) indicate significant differences among the mutants.

The normalized conductance-voltage (G-V, activation) curve of the E1623A showed a slight right shift compared with the WT, with a slower voltage-dependent rising. This is evident as a statistically significant difference in the parameter of the slope of steady-state activation between E1623A and WT (Figure 5A). The depolarized voltage dependence of activation may reduce the availability of the E1623A mutant channels to operate as amplifiers of subthreshold depolarization. The mutant E1623V showed similar alteration, and the other mutants caused slight alterations of activation parameters without statistical significance. Different from a continuity of variations in the steady-state activation curves, the variants were presented as two distinct groups in fast inactivation property (Figure 5B). The steady-state inactivation curves of the mutants E1623A, E1623K, E1623Q, and E1623V were almost overlapped and significantly shifted in the hyperpolarizing direction, whereas the mutants E1623D and E1623G did not differ from the WT. Statistical analysis evidenced that there were two significant subsets on the potential of half-inactivation, but without difference in the slope.

Recovery from inactivation was assessed by using a standard two-pulse protocol. All examined mutants recovered fully within 1 s in this experiment. The fractional recoveries of Na_v_1.1 peak current were plotted against the intervals on a logarithmic time scale. The WT and mutant channels exhibited significantly different kinetics (Figure 5C). Statistical analysis of the time constants (τ-fast and τ-slow) revealed that the E1623A mutant channel recovered from inactivation at the slowest rate among the mutants and was exclusively different from the WT. The E1623A mutant also differed significantly from the E1623D, E1623Q, E1623K, and E1623G mutants in τ-fast.

Taken together, the epilepsy-associated mutant E1623A had significant alterations in all of the four aspects of electrophysiological channel properties and presented the most apparent alteration in three of the four aspects when compared with the other potential mutants. E1623V most closely resembled E1623A, with a reduction in channel conductance and availability during activation and inactivation. E1623Q and E1623K had significant hyperdepolarization shifts only in the voltage-dependent inactivation. No significant alteration was found in either E1623D or E1623G.

### Relationship Between Residue Properties and Electrophysiological Parameters

We analyzed the correlation between amino acid properties and functional alterations of Na_v_1.1 channel (Table 1). The physicochemical properties including molecular size, charge, and polarity were parameterized by their mass, isoelectric point, and hydrophobicity scale, respectively (Supplementary Data); and the ensemble molecular difference was assessed by the established matrices. As shown in Table 1, the hydrophilicity scale was significantly correlated with the channel voltage-dependent activation (slope of activation, *r* = 0.770, *P* < 0.05) and the recovery (slow recovery, *r* = 0.870, *P* < 0.05) parameters. We did not find any significant contributor to current density, which is closely related to the pathogenicity of the mutants. No quantitative correlation between residue properties and inactivation was found, either (Table 1).

**Table 1:** Correlation coefficients between residue similarity and functional changes.

				**Recovery**	
Indices for AA differences	Conductance Peak current	Activation slope	Inactivation V_1/2_	τ-fast	τ-slow
**Simple property**
Volume	0.114	0.394	−0.376	0.544	0.353
Charge	0.361	0.274	0.556	0.018	−0.140
Polarity	0.482	0.770*	0.290	0.705	0.870*
**Physicochemical distance**
Miyata’s distance	0.258	0.599	0.039	0.577	0.626
Grantham’s distance	0.392	0.720	0.083	0.688	0.730
Sneath’s index	0.406	0.634	0.198	0.554	0.413
Experimental exchangeability	0.079	0.140	−0.654	0.439	0.249

Absolute deviations of the average values of electrophysiological parameters between mutants and WT were analyzed for correlations with substitution indices. Electrophysiological parameters with statistical significance to WT were included. The single-pass algorithm of the Pearson correlation coefficient was used to estimate the individual contributions of physicochemical property to specific functional output and the predictive accuracy. Basic physicochemical properties were parameterized by molecular weight, isoelectric point, and hydrophobicity. The hydrophobicity scale of Kyte and Doolittle was adopted as the values of hydrophobicity (Kyte and Doolittle, 1982). *Significant correlation, *P* < 0.05.

**Figure 6:**
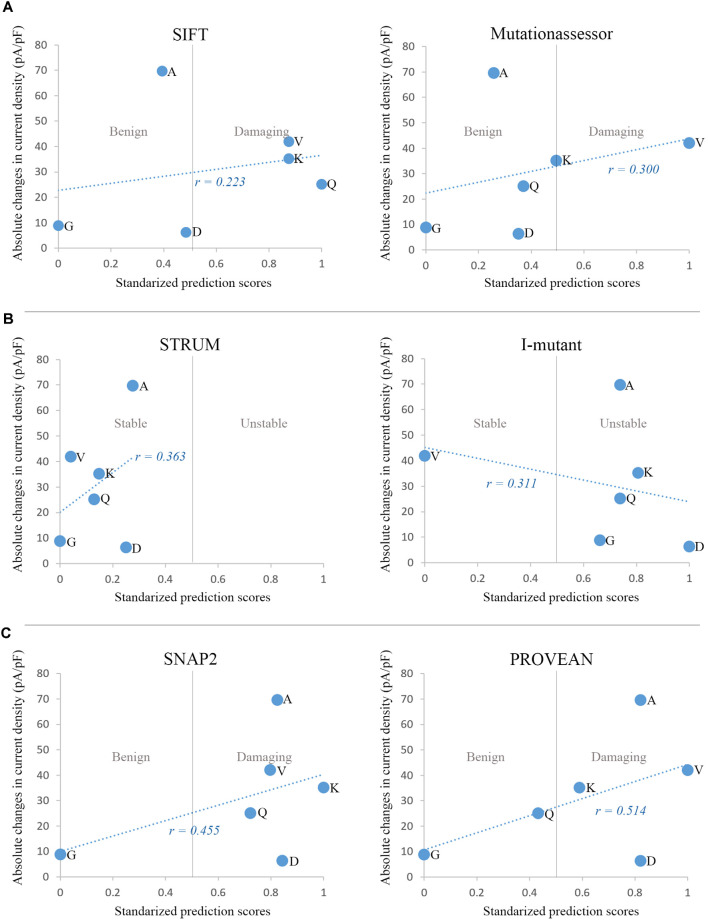
Correlations between in silico prediction scores and functional changes in current density. **(A)** Sequence and evolutionary conservation-based methods: SIFT and Mutationassessor. **(B)** Prediction of protein stability changes by Strum and I-mutant suite. **(C)** Machine-learning methods combining comprehensive features: SNAP2 and PROVEAN. For comparison, prediction scores of the six variants from different tools were normalized to the Polyphen-2 scoring scheme, with a range from 0 to 1 and a threshold at 0.5. The minimal score with benign prediction was set as 0, and the maximal score with damaging prediction was set as 1. Scores with benign prediction were scaled to a range from 0 to 0.5, while scores of damaging variants were scaled to a range from 0.5 to 1.

We analyzed correlations between the predictive scores and alteration of current density. Absolute changes in average current density of the six variants at E1623 were plotted against the normalized predictive scores obtained by different in silico tools, including evolutionary conservation-based methods (e.g., SIFT and Mutationassessor), structure-based (protein stability) predicting tools (e.g., Strum and I-mutant suite), and machine-learning methods that combined comprehensive information (e.g., SNAP2 and PROVEAN; Figure 6). The predictive scores from SNAP2 and PROVEAN appeared to be matched better than other predictive scores (*r* = 0.486 and *r* = 0.485, respectively). However, these predictive scores did not correlate well with the alterations in current density in general.

## Discussion

The sodium channel plays a critical role in controlling the excitability of cells. Na_v_1.1 appears to be a particularly important molecule since it is frequently associated with human diseases (Meisler and Kearney, 2005). Our previous study shows that Na_v_1.1 is highly sensitive to genetic variations (Chen et al., 2015) and thus may be used as a model or an example to explore molecular bases underlying the functioning of ion channels. The present study identified a novel epilepsy-associated mutation E1623A within the extracellular S3-S4 loop of DIV, a previously unidentified functional component. Functional studies demonstrated remarkable loss-of-function of E1623A mutant, with characteristic alterations in activation, inactivation, and recovery properties, and confirmed the pathogenicity of E1623A. More importantly, the other substitutions at the residue E1623 also resulted in apparent functional alterations, suggesting that the residue E1623, presumably as well as the adjacent residues within the extracellular loop, play a critical role in channel gating.

Previous studies have demonstrated that missense mutations in the central pore region of Na_v_1.1 were exclusively associated with loss-of-function, while mutations in the other regions were associated with diverse functional alterations (Meng et al., 2015). In the present study, the novel epilepsy-associated mutation E1623A is located at the extracellular loop linking S3 and S4 in DIV. The roles of extracellular components, especially the loops that connect the transmembrane segments, in the function of Na_v_1.1 channel complex are largely unknown. Based on topological modeling, this extracellular S3-S4 loop appears to be at a considerable distance from the central pore, and is theoretically unlikely to result in a functional change. However, both the severe clinical phenotype in our cases and the existing clinical variants within the loop indicated that the E1623 might be critical for proper channel function.

The subsequent functional studies revealed that the E1623A variant resulted in surprising abnormalities in gating properties, including channel conductance and gating kinetics. Several other potential missense mutants at E1623 also led to changes in gating properties, although to a lesser extent. It is quite interesting that the substitutions in a residue at the extracellular loop that is far apart from the central pore, lead to an overwhelming alteration in channel gating. Since E1623 is located a distance away from the central pore and appears not directly involved in ion conduction, other unknown mechanisms such as electrostatic interaction and local conformational flexibility may be involved. It has been established that sodium currents are elicited in response to changes in the membrane potential sensed by the channel voltage sensor module (S1-S4) containing the S3-S4 linker. Recent studies have uncovered intricate interactions of these elements within the Na_v_ channel signaling complex (Okamura et al., 2015; Catterall et al., 2017). The positive charges in the S4 segments serve as gating charges and move across the membrane electric field when activated, initiating conformational changes to open and close the channel (Yarov-Yarovoy et al., 2012). Upon opening, the upper part of the S4 approaches the pore domain. The S3-S4 loop is lying on the top of the S1-S4 bundles, extending into the extracellular space (Figure 1). Such location suggests that S3-S4 loops may confer local conformational flexibility and thereby impact channel functioning. In a previous study on small-conductance calcium-dependent potassium, a 3-amino acid motif within the extracellular S3-S4 loop was suggested to determine apamin blocking effects and regulate the allosteric change of gating pore (Weatherall et al., 2011). Here multiple sequence alignments of the S3-S4 loop in DIV showed a conserved residue cluster of negative charged residues glutamate (E_XX_E) among Na_v_ superfamily members and negatively charged residues glutamate or aspartate (ExxE/DxxE) in other voltage-gated sodium channels (Figure 2). A similar sequence is present in the S3-S4 loops of different domains. The ExxE forms the tight turn of the loop (Figure 1) and is located close to the voltage-sensor. Therefore, the ExxE motif and the negatively charged acidic glutamate may be crucial for channel functioning, implying that the molecular sub-regional effect should be considered in evaluating the pathogenicity of variants.

It has been demonstrated that mutations of charged residues in S4 of all domains affected activation, whereas those in S4 segments of primarily DI and DIV had the most effects on fast inactivation (Groome and Winston, 2013). Similarly, charge-reversing mutations in S1-S3 segments alter sodium channel activation and fast inactivation, suggesting that positive charge movement across the bilayer must be facilitated by negative charges in other parts of the protein (Chowdhury and Chanda, 2012). In the voltage-gated potassium channels, similar conserved negatively charged residues in VSD have been shown to actively catalyze the transport of ionized groups through the cell membrane and control the voltage sensor operation (Starace and Bezanilla, 2004; Pless et al., 2011; Lacroix et al., 2014). These negatively charged residues are proposed to act as neutralization and electrostatic interactions with positively charged S4 residues, and thus have profound effects on activation gating (Papazian et al., 1995; Yang et al., 2009). Previously, no experimental evidence has been obtained on the role of the extracellular S3-S4 loop in sodium channel functioning. In the present study, the charge-reversing mutation E1623K led to significant changes in fast inactivation with a shifting of steady-state inactivation to more hyperpolarized potentials. In contrast, the negative charge-retaining mutant E1623D showed no alteration in inactivation and was mostly close to WT in fast inactivation parameters (Figure 5). These findings suggest that the negative charge property of E1623 is critical in the inactivation of the sodium channel. Note that the sequence in the N-terminal part of the loop just after S3 is MFLAEL. The residues M, L, and A have high helix propensities. Replacing the E with an A may change the helix-loop-helix structure, and the S3 helical extension into the S3-S4 loop is quite likely to have some effects on the voltage-responsiveness of the S4 helix that could affect both channel activation and inactivation as observed experimentally.

Several mutants without charge-reversing or helix transformation also demonstrated apparent effects on channel activation/inactivation, suggesting much complex relationships between the residue properties of the extracellular loop and channel functioning. We analyzed the correlations between residue properties and electrophysiological alterations. Significant correlations were detected between residue polarity and channel properties (Table 1), indicating that hydrophobicity at the residue E1623 of the extracellular loop is a key property affecting channel functioning. Hydrophobic/polar property is a meaningful scheme clustering residue alphabets. The importance of hydrophobic interaction in protein structure owes much to the following two facts: (i) it is the driving force for protein folding (Dill, 1990); and (ii) it is an important factor for protein-protein interaction (Jones and Thornton, 1996). For hydrophilic glutamate at the extracellular site and on the top of VSDs, its hydrophilic property may play an additional role in favoring the concentration and flow of charged Na^+^ through the lipid bilayer, because extracellular Na^+^ is conducted as a hydrated ion (Payandeh et al., 2012).

However, we did not find any remarkable correlations between the residue properties and peak current density that reflected channel conductance. The present study revealed that the hydrophilic glutamate residue substituted by hydrophobic residues at E1623, such as E1623V and E1623A, did result in decreased current density. However, there were also polarity-altered mutants that did not affect current density, such as E1623G and E1623Q. The confounding factors for the channel conductance at E1623 may include phylogenetic and structural context, together with the elementary changes in amino acid properties. To explore the factors that contributed to channel dysfunction, we used in silico tools to score the mutants from three different perspectives, including sequence/evolutionary conservation, protein stability, and machine-learning protocol with combined comprehensive information and variant dataset of known pathogenicity, based on their working principles. The machine-learning based predictors, SNAP2 and PROVEAN, showed a relatively higher correlation coefficient between the predictive scores and channel conductance.

The conductance of Na_v_1.1 indicated by peak current density is the most crucial property that determines macroscopic gain or loss of channel function. The common form of functional defects caused by genetic variants, identified in epilepsy patients, is loss of function or partial loss of function, featured by non-detectable or reduced sodium current in electrophysiological recordings. Changes in channel conductance determine the pathogenicity of mutations (Tang et al., 2020). In contrast, the electrophysiological parameters of voltage-dependent kinetics of the channel determine the channel characteristics and are associated with mild clinical phenotypes (Liao et al., 2010; Meng et al., 2015). Understanding what residues and functional domains would associate with channel conductance and gating kinetics would provide novel insights into the molecular basis of channel functioning and pathogenicity of variants in channels.

Theoretically, one residue is potentially subject to several different substitutions and subsequently associated with variations in channel function and phenotype, which brings challenges in clinical recognition and diagnosis of genetic disorders. The present study explored the correlation between the nature of substitution and functional alteration in sodium channel Na_v_1.1. It would help understand the intrinsic mechanism underlying the pathogenicity of missense variants. Under the influence of some extrinsic factors, individual cases might exhibit diversity in clinical phenotype and inheritance. In our example, the case 1 and case 2 presented different epileptic phenotypes and inheritance, in which one was severe DS and de novo and the other was epilepsy with febrile seizure plus and dominantly inherited from his mother. In general, a vast majority of de novo severe mutations, especially truncations, were identified in patients with DS, while missense mutations were responsible for familial cases with milder phenotypes (Meng et al., 2015; Liu et al., 2018). This is because de novo mutations are unlikely to pass down to the offspring in patients with severe phenotypes like DS. However, the genotype-phenotype correlation is not entirely consistent. Under some circumstances, the DS-associated mutations, such as the E1623A and a previously reported p.Arg1912X truncation (Jaimes et al., 2020), might exhibit milder phenotype, and thus have the opportunity to develop into an inherited disease in pedigree. The variable disease expressivity might come from individual differences from the modulation of channel physiology, such as trafficking, posttranslational modifications, and pharmacological modulators. It remains a challenging conundrum for researchers about how biophysical alterations in a mutant Na_v_1.1 determine their phenotypes.

Although there has been great progress in genetic sequencing and in silico prediction, variant interpretation remains a major challenge. The present study proposed not only an essential role of the extracellular S3-S4 loop at domain IV in channel functioning, but also an urge of customized predictive tools with more considerations, including 3D structure information, experimental data, and sub-regional function, etc. Different channel functional properties, such as channel conductance and voltage-dependent kinetics, are potentially affected by the distinct molecular alterations and associated with different clinical phenotypes, providing new perspectives on molecular mechanisms underlying genetic diseases.

## Data Availability Statement

The original contributions presented in the study are included in the article/Supplementary Materials, further inquiries can be directed to the corresponding author.

## Ethics Statement

The studies involving human participants were reviewed and approved by Ethics Committee of the Second Affiliated Hospital of Guangzhou Medical University. Written informed consent to participate in this study was provided by the participants’ legal guardian/next of kin. The animal study was reviewed and approved by Ethics Committee of the Second Affiliated Hospital of Guangzhou Medical University. Written informed consent was obtained from the individual(s), and minor(s)’ legal guardian/next of kin, for the publication of any potentially identifiable images or data included in this article.

## Author Contributions

W-PL performed the conceptualization and funding acquisition. W-PL, TS, and M-LC designed research. TS, M-LC, HM, L-HL, BT, X-RL, and W-PL performed research and/or analyzed data. TS wrote the manuscript draft. W-PL contributed to critical manuscript revisions. All authors contributed to the article and approved the submitted version.

## Conflict of Interest

The authors declare that the research was conducted in the absence of any commercial or financial relationships that could be construed as a potential conflict of interest.

## Publisher’s Note

All claims expressed in this article are solely those of the authors and do not necessarily represent those of their affiliated organizations, or those of the publisher, the editors and the reviewers. Any product that may be evaluated in this article, or claim that may be made by its manufacturer, is not guaranteed or endorsed by the publisher.

## Footnotes

^1^ http://scn1a.caae.org.cn/

^2^ http://www.ncbi.nlm.nih.gov/

^3^ https://www.rcsb.org/structure/7DTD

^4^ http://swissmodel.expasy.org
