# Supplementary material for: Critical Role of E1623 Residue in S3-S4 Loop of Nav1.1 Channel and Correlation Between Nature of Substitution and Functional Alteration
Source: Front Mol Neurosci. 2022 Jan 10;14:797628. doi: 10.3389/fnmol.2021.797628 (PMC8785683; doi:10.3389/fnmol.2021.797628)
Supplement: Supplementary file 1 [file Data_Sheet_1.docx]

# Supporting Figure


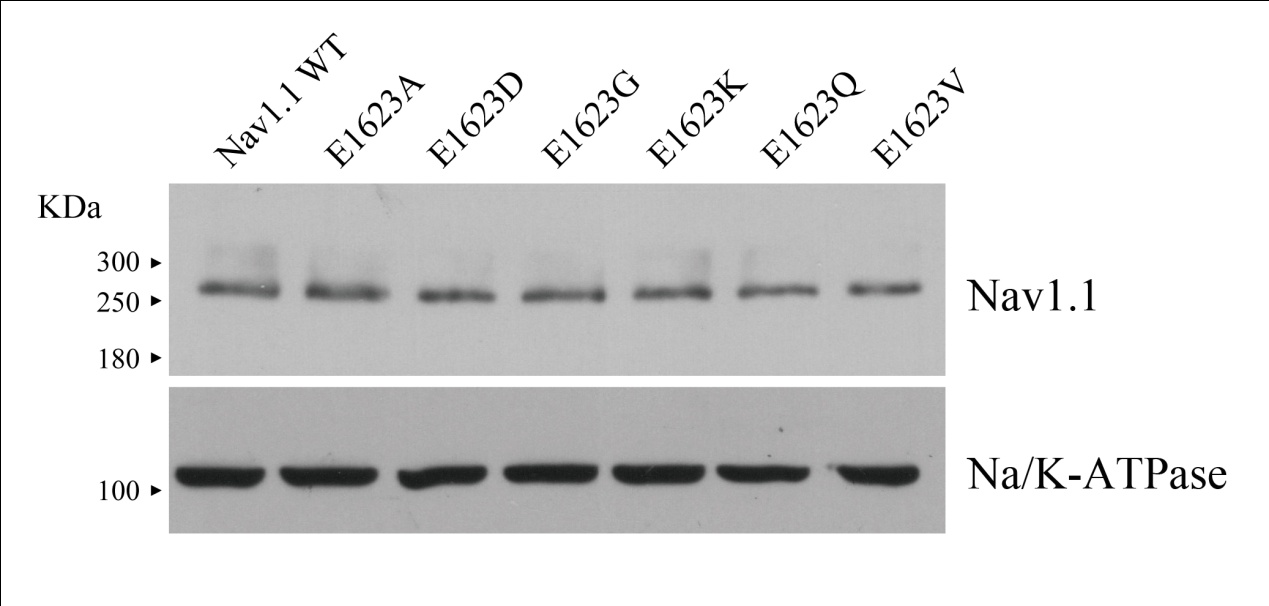


**Supplementary Figure** Membrane expression is not affected by different Na_v_1.1 variants at position 1623.

*Brief methods: Cells were homogenized in lysis buffer 3 days after transfection, subsequently with the isolation of membrane protein fractions using the Plasma Membrane Protein Extraction kit (abcam, ab65400) according to manufacturer's protocol. Protein levels of the mutant Na_v_1.1 on plasma membrane was determined by Western blot using monoclonal mouse anti-Na_v_1.1 antibody. Protein detection was achieved using an appropriate secondary antibody linked to horseradish peroxidase and an enhanced chemiluminescence system.*

# Supporting Table

**Supplementary Table 1** Clinical information of the E1623A variant carriers.

| **Case** | Pedigree | Sex | Diagnosis | Seizure Onset age | Clinical description |
| --- | --- | --- | --- | --- | --- |
| **1** | proband | M | DS | 8 m | The onset was (GTCS) which occurred 1-2 times per month. Subsequently, he suffered from daily myoclonic seizures and atypical absence seizures. He occasionally had status epilepticus triggered by fever. He had normal delivery after a full-term pregnancy. His developmental milestones were normal before one and half years old. Speech delay and intellectual disabilities were observed at the age of two years. Brain MRI scan at 4 years old was normal. The long-term EEG showed generalized high amplitude sharp and slow waves, and focal spike and slow waves in left frontal and temporal region. Topiramate, valproic acid and clonazepam were administrated and reduced the seizures. Lamotrigine, oxcarbazepine and carbamazepine had ever aggravated seizures. He had no family history of epilepsy and febrile seizure. |
| **2** | proband | M | EFS+ | 7 m | He presented his first febrile GTCS at the age of 7 months. Since then, he had febrile or afebrile partial seizures secondary GTCS at the frequency of 2-3 times per year. He had normal delivery and development. He is currently studying in the mainstream middle school with poor academic performance. Brain MRI scan at the age of 14 year were normal. The long-term EEG was normal under the treatment of valproic acid at 600mg/day. He has no seizure for 2 years. |
|  | mother | F | FS | < 6 y | several febrile seizures before six years old |
|  | sister | F | FS | < 6 y | several febrile seizures before six years old |

DS, Dravet syndrome; EFS+, Epilepsy with febrile seizures plus; FS, febrile seizure; GTCS, generalized tonic-clonic seizure;

**Supplementary Table 2** Basic parameters of physiochemical properties of amino acids

| Properties | E | K | Q | D | V | G | A |
| --- | --- | --- | --- | --- | --- | --- | --- |
| Molecular weight (kD) | 147.13 | 146.19 | 146.15 | 133.1 | 117.15 | 75.07 | 89.09 |
| Isoelectric point | 3.15 | 9.6 | 5.65 | 2.85 | 6 | 6.06 | 6.11 |
| kdHydrophobicity | 3 | 2 | 3 | 3 | 16 | 10 | 11 |
